# Cell‐free epigenomes enhanced fragmentomics‐based model for early detection of lung cancer

**DOI:** 10.1002/ctm2.70225

**Published:** 2025-02-05

**Authors:** Yadong Wang, Qiang Guo, Zhicheng Huang, Liyang Song, Fei Zhao, Tiantian Gu, Zhe Feng, Haibo Wang, Bowen Li, Daoyun Wang, Bin Zhou, Chao Guo, Yuan Xu, Yang Song, Zhibo Zheng, Zhongxing Bing, Haochen Li, Xiaoqing Yu, Ka Luk Fung, Heqing Xu, Jianhong Shi, Meng Chen, Shuai Hong, Haoxuan Jin, Shiyuan Tong, Sibo Zhu, Chen Zhu, Jinlei Song, Jing Liu, Shanqing Li, Hefei Li, Xueguang Sun, Naixin Liang

**Affiliations:** ^1^ Department of Thoracic Surgery Peking Union Medical College Hospital Chinese Academy of Medical Sciences and Peking Union Medical College Beijing China; ^2^ Department of Thoracic Surgery Affiliated Hospital of Hebei University Baoding China; ^3^ Shanghai Weihe Medical Laboratory Co., Ltd Shanghai China; ^4^ Department of Cardiothoracic Surgery the Sixth Hospital of Beijing Beijing China; ^5^ Department of Scientific Research Affiliated Hospital of Hebei University Baoding China

**Keywords:** cell‐free DNA, early cancer screening, epigenomics, liquid biopsy, lung cancer

## Abstract

**Background:**

Lung cancer is a leading cause of cancer mortality, highlighting the need for innovative non‐invasive early detection methods. Although cell‐free DNA (cfDNA) analysis shows promise, its sensitivity in early‐stage lung cancer patients remains a challenge. This study aimed to integrate insights from epigenetic modifications and fragmentomic features of cfDNA using machine learning to develop a more accurate lung cancer detection model.

**Methods:**

To address this issue, a multi‐centre prospective cohort study was conducted, with participants harbouring suspicious malignant lung nodules and healthy volunteers recruited from two clinical centres. Plasma cfDNA was analysed for its epigenetic and fragmentomic profiles using chromatin immunoprecipitation sequencing, reduced representation bisulphite sequencing and low‐pass whole‐genome sequencing. Machine learning algorithms were then employed to integrate the multi‐omics data, aiding in the development of a precise lung cancer detection model.

**Results:**

Cancer‐related changes in cfDNA fragmentomics were significantly enriched in specific genes marked by cell‐free epigenomes. A total of 609 genes were identified, and the corresponding cfDNA fragmentomic features were utilised to construct the ensemble model. This model achieved a sensitivity of 90.4% and a specificity of 83.1%, with an AUC of 0.94 in the independent validation set. Notably, the model demonstrated exceptional sensitivity for stage I lung cancer cases, achieving 95.1%. It also showed remarkable performance in detecting minimally invasive adenocarcinoma, with a sensitivity of 96.2%, highlighting its potential for early detection in clinical settings.

**Conclusions:**

With feature selection guided by multiple epigenetic sequencing approaches, the cfDNA fragmentomics‐based machine learning model demonstrated outstanding performance in the independent validation cohort. These findings highlight its potential as an effective non‐invasive strategy for the early detection of lung cancer.

**Keypoints:**

- Our study elucidated the regulatory relationships between epigenetic modifications and their effects on fragmentomic features.
- Identifying epigenetically regulated genes provided a critical foundation for developing the cfDNA fragmentomics‐based machine learning model.
- The model demonstrated exceptional clinical performance, highlighting its substantial potential for translational application in clinical practice.

## Graphical abstract

Lung cancer‐related changes in fragmentomic features were unevenly distributed across the genome, showing significant enrichment in epigenetically regulated regions of specific genes.

The cfDNA fragmentomics‐based model demonstrated superior detection capability, especially for early‐stage lung cancer.

This study offers novel insights into cfDNA biology and paves new avenues for the accurate and cost‐effective early detection of lung cancer.

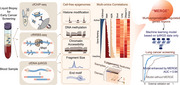

## 1 INTRODUCTION

Lung cancer is one of the leading causes of cancer mortality worldwide.^1^ One of the main reasons is that approximately 75% of lung cancer patients are diagnosed at the advanced stage, resulting in a 5‐year survival rate of less than 10%.^2^ In contrast, early‐stage lung cancer dramatically improves the 5‐year survival rates to 68–92%, underscoring the critical importance of early detection and timely intervention.

While low‐dose computed tomography (LDCT) screening is effective for early lung cancer detection and reducing mortality,^3^ its high false‐positive rate often leads to unnecessary psychological distress and radiation exposure during the follow‐up procedure. Therefore, there remains an urgent need for more precise and non‐invasive screening methods for lung cancer, particularly those targeting early‐stage detection. Liquid biopsy techniques including the analysis of circulating tumour cells (CTCs), circulating tumour DNA (ctDNA) and exosomes, have emerged as promising non‐invasive alternatives in the early detection of lung cancer.^4^ However, their clinical translation is hindered by the challenge of accurately capturing these biomarkers due to the typically low abundance, particularly in early‐stage lung cancer.^5^

Cell‐free DNA (cfDNA), released during cell apoptosis, necrosis and secretion processes, carries abundant epigenomic molecular signatures that make it an ideal biomarker for cancer detection.^6^ For instance, the fragmentation profiles of cfDNA, including fragment size, genomic distribution, breakpoint locations and end motifs, provide insights into nucleosome positioning, chromatin structure and nuclease activity during cell death.^7^, ^8^, ^9^, ^10^ Patients with cancer exhibit distinct cfDNA profiles, characterised by a higher proportion of shorter fragments and a reduced preference for C‐end motifs compared with healthy controls.^11^ Leveraging these fragmentomic features, machine learning models have shown strong performance in detecting advanced lung cancer^12^ and hold promise for multi‐cancer early detection.^13^ Despite this potential, the sensitivity in detecting early‐stage lung cancer, especially stage I cases that would most benefit from early detection and intervention, remains unsatisfactory.^14^ This highlights an urgent need for more accurate approaches to analyse fragmentomic features.

Recent studies have confirmed a strong link between fragmentomic patterns and gene expression in the cells of origin.^15^, ^16^, ^17^ Since the gene expression landscape of cancer cells cannot be directly measured from blood, a promising strategy to enhance diagnostic performance is integrating fragmentomic data with various epigenomic information, which is encapsulated by cell‐free nucleosome complexes. Cell‐free epigenomic features like CpG DNA methylation,^18^, ^19^ histone modifications^20^, ^21^ and chromatin accessibility, particularly in nucleosome‐depleted regions (NDR) at transcription factor binding sites,^22^ are recognised as valuable markers for early cancer detection. Hypermethylation of CpG island on promoters and modification states of histone are sufficient to alter cell fate and result in cancer tumourigenesis, even in the absence of driver mutations.^23^ Plasma cfDNA histone modifications have facilitated the non‐invasive profiling of lung cancer transcriptomes, demonstrating concordant enrichment profiles across various lung cancer subtypes.^24^, ^25^ H3K4me3, a common histone modification, is predominantly enriched at promoters and exhibits a strong positive correlation with transcriptional activity.^20^ Furthermore, the induction of NDRs by transcription factor binding is a critical mechanism for regulating gene expression and modulating chromatin structure.^26^, ^27^ Therefore, integrating these epigenomic layers with fragmentomic features could provide deeper insights into cfDNA‐derived cancer signals and facilitate the development of more accurate detection models.

The detection performance of integrated models has outperformed single‐fragmentomic approaches in hepatocellular carcinoma^28^ and early‐stage breast cancer.^29^ However, it is noteworthy that only a limited number of epigenomic layers were included in these studies, and research on early detection of lung cancer remains scarce. Furthermore, the development of lung cancer, particularly adenocarcinoma, is recognised as a continuous progression from atypical adenomatous hyperplasia (AAH) to adenocarcinoma in situ (AIS), minimally invasive adenocarcinoma (MIA) and ultimately to invasive adenocarcinoma (IAC).^30^ The dynamic changes in epigenetic modifications and fragmentomic features of cfDNA during this process are also highly worth exploring.

To address this gap, we conducted a multi‐centre prospective cohort study to integrate multiple epigenomic layers with diverse fragmentomic features and establish an accurate model for early detecting lung cancer. The epigenomic landscape of plasma cfDNA was characterised using chromatin immunoprecipitation sequencing (cfChIP‐seq), reduced representation bisulphite sequencing (cfRRBS) and low‐pass whole‐genome sequencing (lpWGS). These three approaches target the same molecular entity, the cell‐free nucleosome complex and offer a synergistic framework to explore their crosstalk, an area that remains underexplored. This study aims to provide novel insights into cfDNA biology and open new avenues for early detection of lung cancer.

## 2 METHODS

### 2.1 Study design and participants enrolment

This is a multi‐centre, prospective, cohort study. Participants with suspicious malignant lung nodules, and healthy volunteers in the Affiliated Hospital of Hebei University (AHHU; training cohort) and the Peking Union Medical College Hospital (PUMCH; validation cohort) were consecutively enrolled from November 2022 to December 2023.

Inclusion criteria for individuals with suspicious malignant lung nodules included: (1) age 18 years or older; (2) underwent surgery or biopsy to obtain a definitive pathological diagnosis; (3) able to provide the written informed consent and qualified blood samples. Exclusion criteria included: (1) history of cancer; (2) received anti‐cancer therapy prior to the blood sampling; (3) multiple primary lung cancer. For the age‐ and sex‐matched healthy volunteers, participants were required to meet the following criteria: be at least 18 years old, have no history of cancer and exhibit no indications of suspicious malignant lung nodules based on chest CT screening, which was carefully evaluated by two experienced clinicians. The corresponding demographic and clinical information of the participants was collected and used for the subsequent analyses. Tumour stages were determined according to the eighth edition of the American Joint Committee on Cancer classification.

The study was conducted in accordance with the Declaration of Helsinki and approved by the ethics committee of AHHU (Approval No. HDFYLL‐IIT‐023‐005) and PUMCH (Approval No. I‐23PJ1205). Written informed consent was obtained from all enrolled participants prior participation.

### 2.2 Collection and preparation of samples

Peripheral blood (10 mL) was collected by venipuncture from each subject in Cell‐Free DNA BCT tubes (Streck). Plasma was separated within 2 h by centrifugation at 1600×*g* for 10 min, followed by a second centrifugation at 16 000×*g* for 10 min at 4°C. Haemolysed samples or samples with insufficient material could not be assayed and were excluded. One millilitre aliquots of plasma was stored at −80°C for cfChIP‐seq until analysis. cfDNA was extracted from plasma using the QIAamp Circulating Nucleic Acid Kit (Qiagen) according to the manufacturer's instructions. cfDNA concentration and size distribution were assessed by Qubit fluorometer (Invitrogen; Q33230) and LabChip GX Touch DNA High Sensitivity Assay (PerkinElmer: CLS140158). Quantified cfDNA was stored at −20°C for cfRRBS and lpWGS.

### 2.3 Library preparation for cfChIP‐seq, cfRRBS and lpWGS

For nucleosome capture cfChIP‐seq, 200 µg of H3K4me3 Recombinant Polyclonal Antibody (Invitrogen; 711958) were conjugated to 20 mg of epoxy M270 Dynabeads (Invitrogen; 14301) according to manufacturer's instructions. The antibody covalently conjugated beads were washed and resuspended in PBS containing 0.01% azide preservative at 30 mg/mL and stored at 4°C for use on the same day. In 1 mL of plasma thawed in a water bath, 6.6 µL of antibody covalently conjugated beads were added, along with 1× protease inhibitor cocktail (Roche; 4693132001) and 10 mM EDTA. The reaction was mixed by rotating overnight at 4°C. The beads were magnetised and washed eight times with 200 µL of blood wash buffer (50 mM Tris–HCl, 150 mM NaCl, 1% Triton X‐100, 0.1% sodium deoxycholate, 2 mM EDTA, 1× protease inhibitor cocktail) and three times with 150 µL of 10 mM Tris pH 7.4 on ice. The beads were resuspended in 50 µL of chromatin elution buffer (10 mM Tris pH 8.0, 5 mM EDTA, 300 mM NaCl, 0.6% SDS, 2.5 µL of NEB proteinase K) and incubated for 1 h at 55°C. After magnetisation, the supernatant containing cfDNA was purified using 1.8× Agencourt AMPure XP beads (Agencourt). Double strand library construction of cfDNA was processed by NEBNext Ultra II End Prep kit, NEBNext Ultra II Ligation Master Mix and NEBNext Ligation Enhancer (NEB). The DNA library was amplified by 15 cycles of PCR and purified using 1× AMPure XP. 10 Gbp DNA sequencing data were obtained using the illumina 150 PE program.

For cfRRBS, 10 ng purified cfDNA was cleaved by MspI restriction enzyme (NEB, R0106L) at CCGG sites. Bisulphite conversion of the adapter‐ligated product was carried out with EZ DNA Methylation‐Lightning Kit (ZYMO, D5030). Library construction for cfRRBS was performed using the protocol described in detail elsewhere with minor modifications.^31^ The converted library was amplified using KAPA HiFi HotStart Uracil + ReadyMix Kit (Roche, 07959079001) for 15 cycles. After 1.3× AMPure beads purification, 150PE sequencing was performed on the illumina machine for 20 Gbp data.

For lpWGS, 5 ng of purified cfDNA was processed using the xGen Prism DNA Library Prep Kit (IDT; 10006202), according to the manufacturer's instructions, and then amplified by seven cycles of PCR. 1.3× AMPure X purified library was quantified by Qubit 1× dsDNA HS and LabChip GX Touch DNA High Sensitivity Assay. 10 Gbp DNA sequencing data were obtained using the illumina 150PE program.

### 2.4 Processing of high‐throughput sequencing data

FASTQ files were subjected to processing using Fastp software (v0.23.4) to remove adapters and sequences with low average sequencing quality. For cfChIP‐seq data, bowtie2 (v2.5.3) and sambamba (v1.0) were used for mapping and deduplicating. MACS2 narrow peak BAMPE method (v3.0.1) and bedtools (v2.31.0) were used to call and visualise the enrichment peak. After calculating on‐target reads in consensus peak by featureCounts (v2.18.0), samples with fewer than 0.1 million on‐target reads or an on‐target rate below 30% were excluded. For cfRRBS data, the trimmed reads were aligned to the reference genome hg19 using the Bismark aligner (v0.24.1) and methylation call was performed with methylation extractor script. The results were converted to bedgraph format using Bismark for subsequent analysis. For lpWGS data, the resulting clean data were aligned to the hg19 reference genome using bwa‐mem2 (v2.2.1) and sorted using samtools (v1.17) to obtain positional information for each DNA fragment. PCR‐induced duplicates were removed using sambamba, and reads with low alignment quality, unalignment or unmatched ends were filtered using samtools view with specific criteria (‐f3‐F3852). The remaining DNA fragments were converted to bedpe format using bedtools for subsequent analysis.

### 2.5 Calculation of cfDNA fragmentomic features

We developed in‐house scripts to extract fragmentomic features of cfDNA 6 bp end motif and 2+4 bp breakpoint motif from lpWGS data. The cfDNA 6 bp end motif was determined from the terminal 6‐nucleotide sequence. The cfDNA 2+4 bp breakpoint motif was defined as a 2 bp extension in the 5′ direction and a 4 bp extension in the 3′ direction from the aligned cfDNA 5′ breakpoints within the reference genome. For each sample, we calculated the frequency of each specific motif relative to the total number of motifs, ensuring that the frequencies summed to 1.

The fragmentation size ratio (FSR) was calculated as the proportion of the 151–220 bp fragments within each 1 Mb window relative to the total number of fragments in that window. The fragmentation size distribution (FSD) involved grouping fragments within the 65–400 bp range into 5 bp intervals and calculating the proportion of each group on every chromosome arm.

Transcription factor binding often leads to the formation of NDRs at regulatory regions, influencing local chromatin accessibility and thereby affecting cfDNA fragmentation patterns. To assess this, we calculated the transcription start site (TSS) NDR scores by quantifying the relative depletion of cfDNA fragments within the promoter region, which are known to be strongly associated with transcriptional activity and nucleosome dynamics. Specifically, the relative coverage was calculated as the ratio of the average read coverage within the NDR to the average coverage of its upstream and downstream flanking regions, a measure reflecting the degree of nucleosome depletion at the promoter, as previously reported.^22^ Briefly, for the promoter region (−150 to 50 bp relative to TSS), the mean raw coverage was divided by the mean coverage of upstream (−2000 to −1000 bp relative to TSS) and downstream (1000 to 2000 bp relative to TSS) flanking regions to yield the relative coverage. This normalisation approach was chosen to account for potential biases in sequencing depth and to focus on the relative depletion of nucleosomes in the NDRs.

### 2.6 Gene‐level multi‐omics feature analysis

Using data from the training set, we calculated cell‐free epigenomics and fragmentomics features for each gene based on the UCSC hg19 knownCanonical gene annotation. The H3K4me3 level was quantified using normalised RPKM values within each promoter region. DNA methylation was measured by the mean CpG methylation ratio within each promoter region, derived from cfRRBS data. Fragmentomic characteristics, including the proportions of 0–150 and 151–220 bp fragments, motif proportions and deconvolution contributions, were computed using reads from 1500 bp upstream and the gene body region of each gene. The deconvolution contribution of end‐motifs was calculated by performing a dot product of 4‐mer end motif frequencies with the pre‐trained F‐profile frequencies matrix from Zhou et al.^32^ According to biological research on the six major types of end‐motif components,^32^ in this study, F‐profile I to VI were annotated as DNASE1L3, DNASE1, DFFB, Non‐DNase C‐end, Non‐DNase G‐end and Non‐specific diverse end motifs, respectively. Motif entropy was calculated using 4‐mer Shannon entropy.

For the comparison between cancer and non‐cancer samples, multi‐omics features were averaged within each group, and genes with detectable signals in fewer than 10 samples were filtered out. Cancer‐specific differences were quantified using the *Z*‐score derived from the Mann–Whitney *U* rank sum test. The *Z*‐score was calculated using the formula:

$$\;Z_{\mathrm{score}}=\frac{U_{\mathrm{statistic}}-\frac{\left(n_{1}.n_{2}\right)}{2}}{\sqrt{\frac{n_{1}.n_{2}.\left(n_{1}+n_{2}+1\right)}{12}}}$$

In this formula, *U*_statistic_ refers to the Mann–Whitney *U* statistic, *n*_1_ is the number of cancer samples and *n*_2_ is the number of non‐cancer samples. To improve the performance of rank sum methods for genes with high dynamic range H3K4me3 levels, background correction was applied using the H3K4me3 program from Nir Friedman's cfChIP‐seq software.^20^

### 2.7 Identification and functional analysis of multi‐epigenetically regulated genes

To quantify cfChIP‐seq and cfRRBS data, we utilised the promoter annotations provided by the Ensembl Regulatory Build (GRCh37). Promoter raw count data from cfChIP‐seq were normalised, and differential analysis between lung cancer patients and non‐cancer controls was performed using edgeR software. Meanwhile, the Mann–Whitney *U* test was employed to compare the mean methylation levels of promoter regions obtained from cfRRBS and the NDR scores between the groups.

To capture as many signal differences as possible between patients with lung cancer and non‐cancer controls, we conducted three sets of comparisons: (1) lung cancer versus benign lung nodules; (2) lung cancer versus healthy volunteers; (3) lung cancer versus benign lung nodules and healthy volunteers. Results from the three sets of comparisons were integrated to identify genes regulated by multiple epigenetic modifications in lung cancer. The filtering criteria for multi‐epigenetically regulated gene (MERGE) required that a gene consistently show significant results in at least two differential analyses, with the results being biologically consistent across different omics layers. For instance, significant upregulation in cfChIP‐seq H3K4me3 and significant downregulation in cfRRBS DNA methylation both align with the biological context of gene activation.

The clusterProfiler^33^ tool was utilised to perform gene function and pathway enrichment analysis, encompassing GO Molecular Function, Reactome and WikiPathway databases. Motif enrichment analysis was conducted using the MEME Suite tool in conjunction with the JASPAR2024 CORE non‐redundant database, focusing on regions within ±1 kb of the TSS of genes. Additionally, Genetic Perturbation Similarity Analysis was carried out using the GPSAdb database.^34^

### 2.8 Machine learning model construction and cross‐validation analyses

Fragmentomic features from both the whole genome and MERGE regions were screened for subsequent model training and validation. For MERGE regions specifically, the FSR for each gene interval was calculated as the proportion of 151–220 bp fragments relative to the total number of fragments within that gene. Motif analysis within MERGE regions followed the same methodology as the genome‐wide analysis but was restricted to the defined MERGE intervals.

Initially, each fragmentomic feature was modelled independently to estimate the probability of lung cancer for every participant in the training dataset. The performance of whole genome‐based models and corresponding MERGE‐based models was compared, and the better‐fitting models were selected as the base models for further analysis. Subsequently, an ensemble model was developed by integrating the predicted probabilities from each base model using the Extra Trees algorithm provided by scikit‐learn python library. We then used the ensemble model trained with the best set of hyper‐parameters (1000 for n_estimators, 5 for max_depth and 5 for min_samples_split) for performance measurement. To assess the accuracy of the ensemble model, 10‐fold cross‐validation was performed during the training phase. The calculated score for each participant from the ensemble model was termed the MERGE score, ranging from 0 to 1, with higher scores indicating a greater likelihood of being predicted as lung cancer. After that, the untouched validation dataset was used to evaluate the detection performance of the multi‐dimensional ensembled machine learning model. Due to clinical controversies regarding its true classification, AAH was not incorporated in the formal efficacy evaluation of the ensemble model but was only used for exploratory analyses.

### 2.9 Clinical benefits estimation

To evaluate the clinical utility of the ensemble model, an interception model was introduced^35^ and adopted with epidemiological data on lung cancer,^36^ stage at diagnosis^37^ and 5‐year survival rate^38^ in China. Under the most conservative dwell time scenario (aggressive fast mode), the benefit of cancer stage shifted from late (stage III/IV) to early (I/II) and the improvement of 5‐year survival rate were evaluated at different screening intervals (from 6 months to 5 years). The original code is available at Hubbell_CEBP_Inteerception.

### 2.10 Statistical analysis

All statistical analyses were performed using R version 4.4.0. Continuous variables were described with medians and interquartile ranges and categorical variables with numbers and percentages. The Mann–Whitney *U* rank sum test was used to compare continuous variables between two groups and the Kruskal–Wallis test for multiple groups. Categorical variables were analysed using the Chi‐squared test or Fisher's exact test, as appropriate. Based on true‐positive (TP), true‐negative (TN), false‐positive (FP) and false‐negative (FN) of lung cancer prediction, the sensitivity [TP/(TP + FN)], specificity [TN/(TN + FP)], positive predictive value (PPV) [TP/(TP + FP)] and negative predictive value (NPV) [TN/(TN + FN)] values were calculated. The receiver operating characteristic (ROC) curves were generated using the pROC package. Areas under the ROC curves (AUC) of prediction models were compared using the DeLong test. For the AUC, the 95% confidence interval was computed using 2000 stratified bootstrap replicates. For sensitivity, specificity, PPV and NPV, the Wilson method from the binom package was employed to calculate the corresponding 95% confidence intervals.

## 3 RESULTS

### 3.1 Study overview and cohort characteristics

The cell‐free epigenome landscape is distinct from that in cells. Due to the complex processes of cell death and circulating digestion, as well as the mixture of cfDNA from various sources and digestion stages, cfDNA carries a rich array of versatile epigenetic modifications with unique biases.^39^ We believe that for liquid biopsy based on cfDNA, specifically examining the epigenetic information in cfDNA, should perform better than relying solely on prior knowledge from tissues. Therefore, we hypothesise that cancer screening based on fragmentomics will greatly benefit from systematic analysis of the cell‐free epigenome.

**FIGURE 1:**
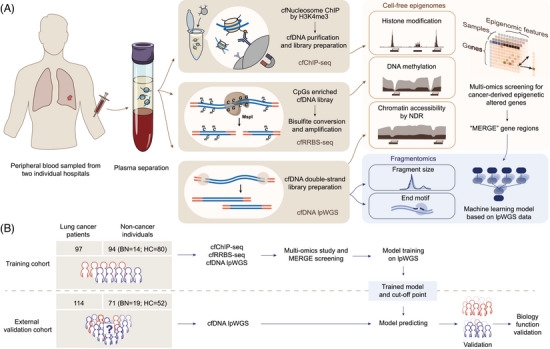
Schematic of overall approach for lung cancer early detection. (A) Diagram illustrating the sequencing, data analysis and modelling methodology. cfDNA extracted from plasma samples underwent cfChIP‐seq, cfRRBS and lpWGS. Cell‐free epigenomic features were extracted and comprehensively analysed. Cancer‐derived epigenetically altered genes were screened based on their cell‐free epigenomic profiles to identify MERGE candidates. Fragment features, including fragment size and end motifs from lpWGS, were analysed to develop a MERGE‐enhanced cancer detection model. (B) Cohorts used for the development and validation of the lung cancer screening model. The training cohort was used for MERGE selection, model training and cut‐off determining. External validation cohort was used for model validation and further biological function research. cfChIP‐seq, cell‐free chromatin immunoprecipitation sequencing; cfRRBS, cell‐free reduced representation bisulphite sequencing; lpWGS, low‐pass whole‐genome sequencing; NDR, nucleosome‐depleted regions; MERGE, multi‐epigenetically regulated genes; BN, benign nodules; HC, healthy controls.

To test this hypothesis, we focused on three types of cell‐free epigenomic characteristics: histone modification, DNA methylation and chromatin accessibility. The study measured cell‐free nucleosome H3K4me3 levels at promoter regions, assessed the CpG methylation status of these regions and examined NDRs induced by transcription factor binding. Specifically, nucleosome depletion refers to the phenomenon in which nucleosomes are removed or less abundant in specific genomic regions, thereby enhancing the accessibility of the underlying DNA to the transcriptional machinery.^40^ By comprehensively investigating the synergistic impact of these types of epigenomic features on fragmentomics, we aimed to classify a series of cancer‐derived MERGEs regions for a fragmentomics‐based cancer screening model (Figure 1A).

A total of 376 participants were included in this study for model construction (AHHU; *n* = 191) and validation (PUMCH; *n* = 185) (Figure 1B). The training cohort included 97 subjects with malignant nodules, 14 with benign nodules and 80 healthy controls, whereas the validation cohort comprised 114 subjects with malignant nodules, 19 with benign nodules and 52 healthy controls. We then stratified all participants into cancer (malignant) and non‐cancer (benign and healthy) groups for subsequent analysis. Among those with lung cancer, the training set included 60 (61.9%) subjects in stage I and 20 (20.6%) in stage II, while the validation set comprised 24 (23.1%) subjects in stage 0, 61 (58.7%) in stage I and 2 (1.9%) in stage II. The detailed clinical characteristics of participants in each cohort were summarised in Tables S1 and S2.

### 3.2 Multiple cell‐free epigenomes synergistically affect fragmentomic features

**FIGURE 2:**
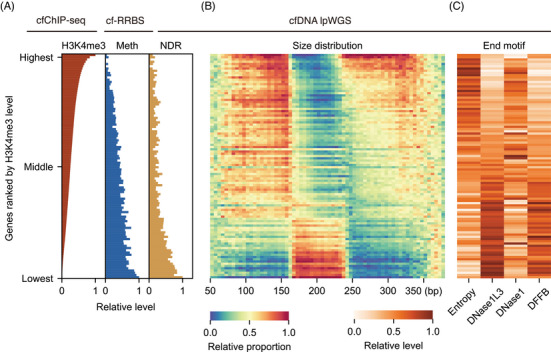
Correlations between multiple cell‐free epigenomes and fragmentomic features in non‐cancer samples. (A–C) Each row corresponds to genes ranked by H3K4me3 levels from highest to lowest based on cfChIP‐seq data and grouped into 100 percentiles. All genes with detectable H3K4me3 peak were include (*n* = 11 479). DNA methylation levels of each gene were measured by the CpG methylation ratio in the TSS ± 1.5 kb region using cfRRBS. The NDR score was determined by nucleosome coverage at the TSS site using lpWGS sequencing. b‐c Heatmap depicting cfDNA fragment size distribution (B) and fragment end motifs (C) on cfDNA lpWGS data. The x axis in b depicts cfDNA fragment size in 5 bp average window. In C, entropy was calculated by 4‐mer end motif. DNase contributions were calculated using the F‐profiles deconvolution matrix with 4‐mer end motif proportions. The colours and bar heights were scaled column‐wise to a range of 0 to 1 to display the relative values. These data were analysed from non‐cancer samples in the training set. Meth, DNA methylation; TSS, transcription start site.

We first depicted the relationships among multiple cell‐free epigenomes. Since H3K4me3 modification, CpG methylation and nucleosome depletion by transcription factor binding are all closely related to gene expression,^22^, ^41^ we focused on gene‐level analysis. We calculated the average epigenomic features in cancer and non‐cancer samples from the training set. All genes were ranked by their H3K4me3 levels, from highest to lowest, and grouped into 100 percentiles. The results showed that as the H3K4me3 level of a gene increased, the DNA methylation and NDR occupancy near the promoter exhibited a consistent decrease (Figure 2A). Our cell‐free epigenomes analysis aligned with cellular studies showing that highly expressed genes exhibit lower promoter methylation and stronger nucleosome depletion (i.e., lower NDR scores).^42^ This clear trend was confirmed in cancer samples as well (Figure S1A).

The dynamic changes in fragmentomics arise from the behaviour of various DNases on different chromatin states during the cell death process, reflecting the influence of epigenetic regulation before cell death.^43^ To elucidate the impact of cell‐free epigenomes on fragmentomics, we computed fragment size and end‐motifs within gene bodies and their 1  kb upstream regions. We included the 1  kb upstream region due to the distinct characteristics observed in the promoter region's fragmentome. In both H3K4me3‐enriched cfDNA and whole‐genome cfDNA, promoter region fragments were found to be shorter than those in the background genome, showing a noticeable left shift in the distribution plot (Figure S2A‐D). This underscored the significance of analysing the upstream region of genes.

After building a single‐gene‐resolution fragment feature matrix, we profiled these features according to the ranking of genes determined by their H3K4me3 levels. In terms of fragment size, we observed that activated genes marked by cell‐free epigenomes (with higher H3K4me3 levels, lower DNA methylation and reduced NDR occupancy) tend to have shorter fragment sizes. For genes with higher upregulated rank, mononucleosomes showed more fragments on the left side of peak (<167 bp) and fewer on the right, while dinucleosomes showed both peak's left enrichment and a gradual leftward shift of peak's summit (Figure 2B). This distribution dynamic indicated that epigenetically upregulated regions undergo more intense digestion for both mononucleosomes and dinucleosomes. Additionally, the observed dinucleosomes peak's summit shift in promoters and activated genes implied a shorter nucleosome spacing. This aligns with previous findings that open chromatin and transcription regions are associated with more intense degradation and shorter nucleosome repeat lengths.^7^

For fragment end motifs, we performed entropy calculation and F‐profiles deconvolution analyses^32^ for each gene in our lpWGS data. This approach allowed us to understand the potential origins during nucleosome release. For epigenetically upregulated genes, the fragments showed a more chaotic motif pattern and significantly fewer DNase1L3 and DFFB‐contributed motifs (Figure 2C), suggesting that upregulated genes were strongly related to the disorganisation of DNA cleavage processes during cell death, making the DNA more likely to form amorphous end motifs.

In lung cancer samples, fragment size and end motifs correlated with H3K4me3 levels in a trend similar to that observed in non‐cancer samples, highlighting the widespread impact of epigenomes on fragmentomics (Figure S1B,C). Moreover, there was no apparent difference in the distribution of fragment size proportions among cancerous, benign and healthy samples (Figure S2E). Therefore, a detailed analysis of fragmentomics at the specific gene level, rather than at the whole genome scale, may provide more valuable information for the accurate detection of lung cancer.

### 3.3 Cancer‐derived fragmentomic changes enriched in epigenetically dysregulated gene hotspots

**FIGURE 3:**
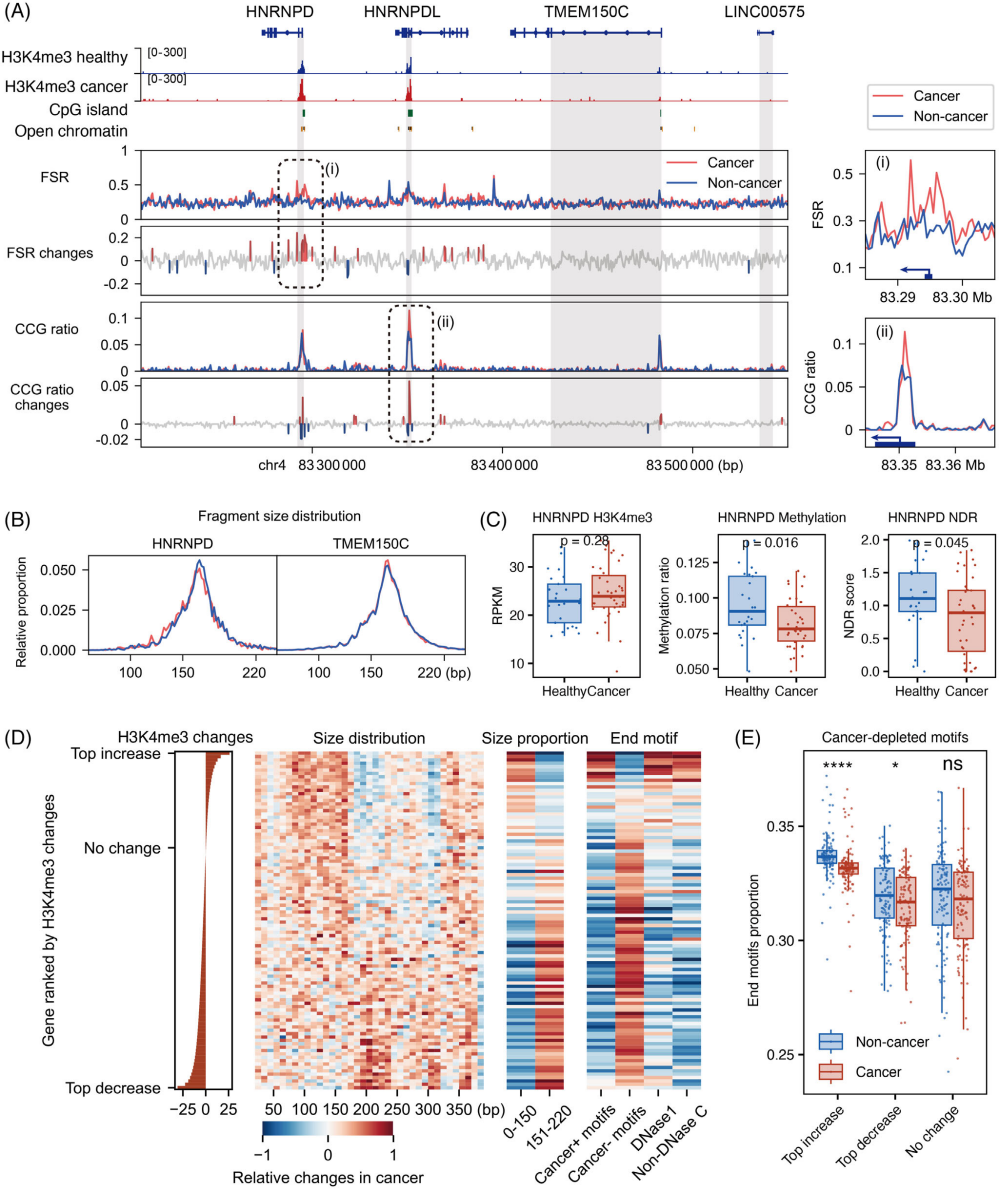
Cancer‐derived fragmentome hotspots and cell‐free epigenome changes. (A) The genomic region of chromosome 4 at q21.22 was shown, with several shaded areas marking the positions of genes from the promoter to the first exon. Epigenetic modifications, including H3K4me3 (cfChIP‐seq data from a healthy individual, P292, and a cancer patient, P318), CpG islands and open chromatin regions (DNase I hypersensitive sites from the A549 lung cancer cell line), were displayed. Fragmentome features, such as the FSR (ratio of 0–150 to 151–220 bp fragments) and the CCG‐end proportion, were calculated using 500 bp bins from all cancer (red) or non‐cancer (blue) samples combined. The differences in these features are also illustrated, with significant bins (>2*σ*) highlighted. A zoom‐in plot of two notable changes, (i) and (ii), is shown on the right. (B) Size distributions in HNRNPD and TMEM150C genes for the two groups. (C) Cell‐free epigenome changes of the HNRNPD gene between groups. (D) Each row represents genes ranked by changes in H3K4me3 levels, from increase to decrease, grouped into 100 percentiles. The heatmap in the middle shows fragment size distribution (10 bp windows). Cancer‐enriched (cancer+) or depleted (cancer‐) 4‐mer motifs were identified with an adjusted *p* value of <.05 between cancer and non‐cancer samples. Colours and bar heights were *Z*‐score scaled column‐wise to a range of −1 to 1 to show relative changes. (E) End motif proportions of cancer‐depleted motifs were calculated for the top 1% of genes with increased, decreased and minimal changes in H3K4me3 levels between cancer and non‐cancer samples. These data were analysed from samples in the training set. Asterisks indicate statistical significance (*p* value were computed with Mann–Whitney *U* rank sum test, ns: *p* > .05, *: *p* < .05, **: *p* < .01, ***: *p* < .001, ****: *p* < .0001).

Recognising that dynamic changes in fragmentomic features are marked by cell‐free epigenomes, we focused on capturing the subtle signals of lung cancer at a high‐resolution scale and exploring their relationship with these epigenomic markers. By combining all non‐cancer and cancer samples in the training set, we were able to analyse fragmentomic features in bins at near‐nucleosome resolution (500 bp). Indeed, fragment size and end motif features showed fluctuations around the H3K4me3 peak, CpG islands and regions of open chromatin (Figure 3A).

Surprisingly, while fragmentomics exhibited minimal cancer‐specific alterations in the majority of genomic regions, significant changes nearly perfectly matched the positions of these multi‐epigenetic modifications. For example, on chromosome 4 at q21.22, significant changes (>2*σ*) appeared at the promoters of the HNRNPD and HNRNPDL genes, aligning with cfChIP‐seq H3K4me3 peak (Figure 3A). However, nearby regions such as the TMEM150C gene and intergenic areas showed very few cancer‐derived changes. We observed a tendency for shorter fragments in the HNRNPD gene region in cancer samples, but not in TMEM150C (Figure 3B). Moreover, HNRNPD was functionally active in cancer samples, characterised by cell‐free epigenomes with upregulated H3K4me3 levels, lower DNA methylation and reduced NDR occupancy (Figure 3C). HNRNPD and its similar gene HNRNPDL have been reported to be strongly upregulated in various cancers, including lung cancer, and play key roles in inducing tumour growth and metastasis,^44^ while TMEM150C is not well studied in cancer biology. These results together suggested that cancer‐specific alterations in fragmentomics were more enriched in genes regulated by epigenetic modifications, rather than being evenly distributed across all genomic regions.

To further investigate these relationships across all genes, we utilised a single‐gene level multi‐omics features matrix to conduct non‐parametric statistical tests, comparing cancer samples with non‐cancer samples in the training set. This allowed us to quantify the direction and magnitude of cancer‐derived changes by calculating a gene‐wide *Z*‐score, estimated using the Mann–Whitney *U* test. We then ranked all genes based on their H3K4me3 changes in cancer and mapped the corresponding fragmentomic changes onto that ranking (Figure 3D).

For cancer‐upregulated genes with increased H3K4me3 levels, we observed a relatively higher distribution of fragments in the 50 to 160 bp range, along with a greater prevalence of cancer‐enriched motifs, DNase1 origin motifs and non‐DNase C‐end motifs (Figure 3D). In contrast, cancer‐downregulated genes with decreased H3K4me3 levels showed a notable increase in size distribution around 200 bp and an abundance of cancer‐depleted motifs (Figure 3D). Statistical comparisons between non‐cancer and cancer samples at the top 1% of genes with H3K4me3 upregulation, downregulation and minimal changes revealed distinct differences in fragmentomic features. (Figures 3E and S3A,B). Similar statistical analyses were performed for cfDNA methylation and NDR rank, confirming these trends (Figure S3B).

These results support our hypothesis that cancer‐specific fragmentomic features are enriched in hotspots near gene promoters and gene bodies, which are epigenetically dysregulated and marked by cell‐free epigenomes. This insight provides a more targeted approach for isolating cancer‐derived signals from background noise.

### 3.4 Identification and characterisation of MERGEs in lung cancer

Our comprehensive analysis of cell‐free epigenomics revealed that cancer‐derived epigenetically regulated genes exhibited more significant fragmentomic characteristics compared with the whole genome, potentially enhancing the precision of lpWGS‐based early cancer screening methods.

**FIGURE 4:**
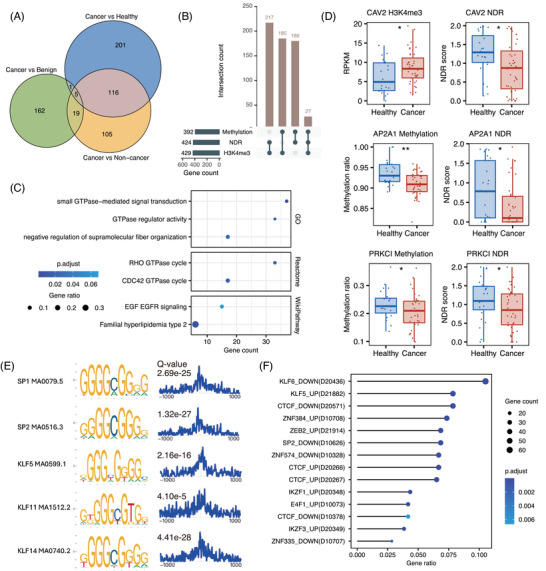
Identification and characterisation of multi‐epigenetically regulated genes. (A) Venn diagram illustrating the overlap of MERGEs identified from three comparative analyses: cancer versus healthy (blue), cancer versus benign (green) and cancer versus non‐cancer (yellow). The numbers represent the unique and shared MERGEs among different comparisons. (B) Intersection of epigenetic modifications in MERGEs. Horizontal bars indicate the total number of genes regulated by each epigenetic modification (methylation, NDR, H3K4me3). Vertical bars show the number of genes co‐regulated by different combinations of these modifications. (C) Functional annotation of MERGEs using GO Molecular Function, Reactome and WikiPathway databases. The *x*‐axis represents the number of overlapping genes, while the *y*‐axis shows enriched functions or pathways. Dot size indicates the proportion of overlapping genes, and colour intensity represents the adjusted *p* value. (D) Epigenetic profiles of CAV2, AP2A1 and PRKCI, representative genes in the EGFR signalling pathway, in healthy and cancer samples. Box plots display H3K4me3 levels (RPKM) and nucleosome‐depleted regions (NDR) scores, with *p* values indicating significant differences between groups. (E) Motif enrichment analysis of MERGEs promoters. Left: sequence logos of five significantly enriched SP/KLF family motifs identified by MEME‐ChIP within ± 1 kb of transcription start sites (TSS). Right: corresponding motif distribution plots showing enrichment frequency relative to TSS. (F) Genetic Perturbation Similarity Analysis (GPSA) of MERGEs. The dot plot displays enriched C2H2 zinc finger transcription factors. The *x*‐axis represents the gene ratio, while the y‐axis shows enriched transcription factors and their regulatory relationships. “UP” indicates gene sets upregulated after transcription factor knockout, while “DOWN” indicates downregulated gene sets. Parentheses contain the corresponding GPSA dataset ID. Dot size indicates the number of overlapping genes, while colour intensity represents the adjusted *p* value. Asterisks indicate statistical significance (*p* value were computed with Mann–Whitney *U* rank sum test, ns: *p* > .05, *: *p* < .05, **: *p* < .01, ***: *p* < .001, ****: *p* < .0001).

To identify these genes, we conducted statistical tests on histone modification, DNA methylation and chromatin accessibility signals across three comparative groups: cancer versus non‐cancer, cancer versus healthy and cancer versus benign (Figure S4). Integrating the results from these comparisons, we identified a total of 609 MERGEs. Specifically, 245 were identified in the cancer versus non‐cancer group, 323 in the cancer versus healthy group, and 187 in the cancer versus benign group (Figure 4A). Among these genes, 217 were co‐regulated by H3K4me3 and NDR, 185 by H3K4me3 and methylation and 180 by methylation and NDR. Additionally, 27 genes exhibited abnormal regulation by all three epigenetic modifications (Figure 4B and Tables S3–S6).

Functional annotation of MERGEs using multiple databases revealed significant enrichment in GTPase‐mediated signal transduction (GO: 0007264, padj = .001; Reactome: R‐HSA‐9013148, padj = .016) and the EGF/EGFR signalling pathway (WikiPathway: WP437, padj = .068) (Figure 4C). Key genes within these pathways, including CAV2, AP2A1 and PRKCI, exhibited significant multi‐omics epigenetic regulatory associations (Figure 4D). These pathways are well‐established contributors to tumour invasion, metastasis and metabolic reprogramming,^45^, ^46^ underscoring the potential role of epigenetic dysregulation in driving their aberrant activation during early‐stage lung cancer.

Motif enrichment analysis within ±1 kb of MERGEs’ TSS revealed significant enrichment of binding motifs for C2H2 zinc finger DNA‐binding proteins, particularly members of the Sp/KLF family (Figure 4E). Corroborating these findings, genetic perturbation similarity analysis demonstrated enrichment of MERGEs regulated by C2H2 zinc finger transcription factors, including SP2, KLF5/6 and CTCF (Figure 4F). These converging lines of evidence suggested that MERGEs are likely subject to transcriptional regulation by the Sp/KLF family through epigenetic mechanisms during early‐stage lung cancer development.

### 3.5 Fragmentomics‐based ensemble MERGE model enables accurate detection of lung cancer

**FIGURE 5:**
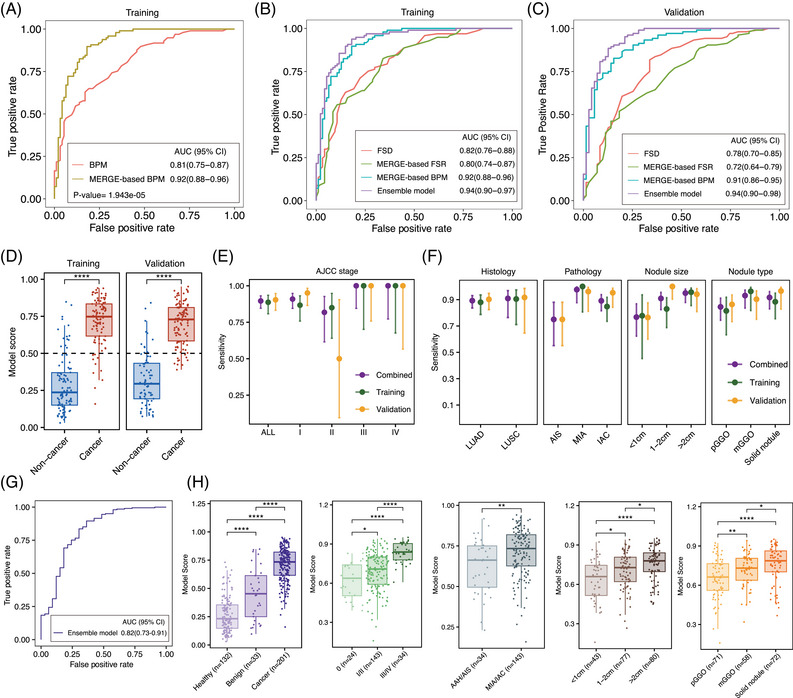
Identification and evaluation of the MERGE‐based ensemble model for lung cancer detection. (A) Receiver operating characteristic (ROC) curve evaluating the performance of BPM and MERGE‐based BPM in distinguishing cancer from non‐cancer subjects in the training set. (B) ROC curve evaluating the performance of the MERGE‐based ensemble model and three base models in the training set. (C) ROC curve evaluating the performance of the MERGE‐based ensemble model and three base models in the validation set. (D) Boxplots showing the distribution of model scores in the training and validation set. (E) Sensitivities of the MERGE‐based ensemble model across different tumour stages. (F) Sensitivities of the MERGE‐based ensemble model across different pathologic and radiological subgroups. (G) ROC curve evaluating the performance of the MERGE‐based ensemble model in distinguishing lung cancer from benign lung nodules in the combined set. (H) Boxplots showing the distribution of model scores across different pathologic and radiological subgroups in the combined set. BPM, break point motifs; FSD, fragmentation size distribution; EDM, end motifs; FSR, fragmentation size ratio. Asterisks indicate statistical significance (*p* value were computed with Mann–Whitney *U* rank sum test, ns: *p* > .05, *: *p* < .05, **: *p* < .01, ***: *p* < .001, ****: *p* < .0001).

The cfDNA fragmentomic features include break point motifs (BPM), MERGE‐based BPM, end motifs (EDM), MERGE‐based EDM, FSR, MERGE‐based FSR and FSD were screened for subsequent model construction. First, the single feature‐based classification performance was compared for BPM, EDM and FSR across the whole genome and the MERGE regions. Overall, the corresponding MERGE‐based models all displayed superior discrimination in the training set as indicated by higher AUCs (Figures 5A and S5A). Moreover, considering that BPM and EDM were similar dimensional features and BPM had better predicative ability, MERGE‐based BPM was retained. Finally, the three single base classifiers, MERGE‐based BPM, MERGE‐based FSR and FSD, were integrated to construct the MERGE‐based ensemble model.

Based on the 3× coverage lpWGS data, the MERGE‐based ensemble model outperformed the three base classifiers and the whole genome‐based ensemble model, achieving an AUC of 0.94 (95% CI: 0.90–0.97) in the training set (Figures 5B and S5B). The locked ensemble model was then independently verified in the external validation set and reached an AUC of 0.94 (95% CI: 0.90–0.98) (Figure 5C). Furthermore, MERGE scores of lung cancer subjects were found to be significantly higher than non‐cancer controls in both the training set and the validation set (Figure 5D and Table S7). Using 0.5 as a cutoff, the ensemble model achieved a sensitivity of 88.7% at the 85.1% specificity in the training set and a sensitivity of 90.4% at the 83.1% specificity in the external validation set, respectively (Table 1). It is especially noteworthy that the ensemble model maintained its sensitivity even in stage I cases (86.7% in the training set, 95.1% in the validation set and 90.9% in the combined set) (Figure 5E). Additionally, the model performance across different pathological and radiological subgroups was further examined (Figure 5F and Table S8). The ensemble model correctly classified 96.2% of MIA and 75% of AIS cases in the validation set and seven out of ten AAH subjects had MERGE scores above the cut‐off value.

**TABLE 1:** The diagnostic performance of the MERGE‐based ensemble model in the training, validation and combined sets.

	Training set *n* = 191	Validation set *n* = 175	Combined set *n* = 366
All			
Sensitivity	0.887(0.808–0.935)	0.904(0.832–0.947)	0.896(0.846–0.931)
Specificity	0.851(0.765–0.909)	0.831(0.727–0.900)	0.842(0.779–0.890)
PPV	0.86(0.779–0.915)	0.887(0.812–0.934)	0.874(0.821–0.912)
NPV	0.879(0.796–0.931)	0.855(0.753–0.919)	0.869(0.808–0.913)
Stage l			
Sensitivity	0.867(0.758–0.931)	0.951(0.865–0.983)	0.909(0.845–0.948)
Specificity	0.851(0.765–0.909)	0.831(0.727–0.900)	0.842(0.779–0.890)
PPV	0.788(0.675–0.869)	0.829(0.724–0.899)	0.809(0.735–0.866)
NPV	0.909(0.831–0.953)	0.952(0.867–0.983)	0.927(0.873–0.959)
MIA			
Sensitivity	1(0.806–1)	0.962(0.811–0.993)	0.976(0.877–0.996)
Specificity	0.851(0.765–0.909)	0.831(0.727–0.900)	0.842(0.779–0.890)
PPV	0.533(0.361–0.698)	0.676(0.515–0.804)	0.612(0.492–0.72)
NPV	1(0.954–1)	0.983(0.911–0.997)	0.993(0.961–0.999)
<1 cm			
Sensitivity	0.778(0.453–0.937)	0.765(0.6–0.876)	0.767(0.623–0.868)
Specificity	0.851(0.765–0.909)	0.831(0.727–0.900)	0.842(0.779–0.890)
PPV	0.333(0.172–0.546)	0.684(0.525–0.809)	0.559(0.433–0.678)
NPV	0.976(0.915–0.993)	0.881(0.782–0.938)	0.933(0.881–0.963)
pGGO			
Sensitivity	0.815(0.633–0.918)	0.864(0.733–0.936)	0.845(0.743–0.911)
Specificity	0.851(0.765–0.909)	0.831(0.727–0.900)	0.842(0.779–0.890)
PPV	0.611(0.449–0.752)	0.76(0.626–0.857)	0.698(0.594–0.785)
NPV	0.941(0.87–0.975)	0.908(0.813–0.957)	0.927(0.873–0.959)

Abbreviations: MIA, minimally invasive adenocarcinoma; NPV, negative predictive value; pGGO, pure ground‐glass opacity; PPV, positive predictive value.

When applying the same cutoff score of 0.50, the ensemble model achieved an AUC of 0.816 (95% CI: 0.725–0.907) and a sensitivity of 89.6% at 60.6% specificity for distinguishing lung cancer from benign lung nodules (Figure 5G). This suggested that the ensemble model can also assist in determining the nature of suspicious malignant lung nodules detected by LDCT screening. Moreover, the model score significantly increased with later stages of disease, more invasive phenotype, larger tumour diameter and higher consolidation/tumour ratio (Figure 5H). Other baseline clinical characteristics, including gender, age, smoking status and heredity, did not exhibit any statistically significant effects on the model scores (Figure S6A). Taken together, these analyses suggested that the fragmentomics‐based ensemble model harboured explicit biological plausibility and was suitable in different clinical scenarios.

In the intended‐use population with a prevalence of malignant nodules at 0.107% in the age group of 40–74 years in China,^36^ the NPV of our model reached as high as 99.9%. This indicated that the ensemble model would not only enhance lung cancer detection but also reduce unnecessary procedures. Furthermore, an adapted interception model was employed to evaluate the clinical value of the ensemble model in real‐world practice.^35^ Under the most aggressive dwell time scenario, the interception model showed that annual screening would shift 81% of advanced lung cancers to early stages at initial diagnosis and improve the 5‐year overall survival rate from 38.80 to 67.47% (Figure S6B and Table S9).

### 3.6 Epigenetic patterns of MERGEs mirror lung adenocarcinoma progression

**FIGURE 6:**
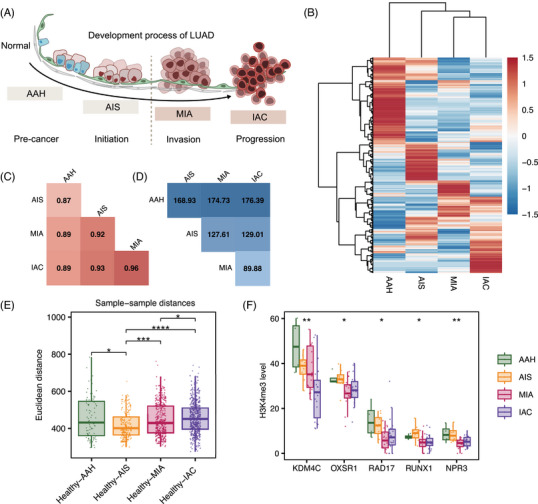
H3K4me3 patterns of multi‐epigenetically regulated genes in lung adenocarcinoma progression. (A) Diagram of the lung adenocarcinoma development process. (B) Unsupervised clustering heatmap of H3K4me3 cfChIP‐seq signals at MERGEs promoters across different pathological stages of lung adenocarcinoma. Each column represents the mean enrichment level (mean rpkm) of all samples within a subtype, and each row represents a gene. The colour scale ranges from red (high) to blue (low) indicating H3K4me3 abundance. (C) Correlation matrix of H3K4me3 levels among lung adenocarcinoma subtypes. Values represent Pearson correlation coefficients. (D) Euclidean distance matrix of H3K4me3 levels among lung adenocarcinoma subtypes. Values indicate calculated Euclidean distances. (E) Box plot showing sample‐to‐sample Euclidean distances of H3K4me3 profiles between healthy controls and each lung adenocarcinoma subtype. (F) Box plots show H3K4me3 enrichment levels (measured as promoter rpkm) for five genes (KDM4C, OXSR1, RAD17, RUNX1 and NPR3R) at different stages of lung adenocarcinoma progression. AAH, atypical adenomatous hyperplasia; AIS, adenocarcinoma in situ; MIA, minimally invasive adenocarcinoma; IAC, invasive adenocarcinoma. Asterisks indicate statistical significance (*p* values were calculated using the Kruskal test, ns: *p* > .05, *: *p* < .05, **: *p* < .01, ***: *p* < .001, ****: *p* < .0001).

The score of the MERGE‐based model increased progressively with the aggressiveness of lung adenocarcinoma (LUAD), suggesting that MERGE may contribute to its development. Considering the evolutionary trajectories from AAH to IAC and the significant impact of epigenetic regulation on tumour development, the associations between H3K4me3 modification patterns of MERGE and LUAD subtypes were further explored (Figure 6A). Unsupervised clustering of H3K4me3 profiles revealed distinct patterns among LUAD subtypes (Figure 6B). Notably, aberrant H3K4me3 modifications were detectable as early as the AAH stage, potentially indicating their role in early carcinogenesis. As the disease progressed from preneoplastic AAH to preinvasive AIS, microinvasive MIA and finally IAC, we observed a gradual increase in correlation and a concurrent decrease in Euclidean distance across the H3K4me3 profiles of different subtypes (Figure 6C,D). Of these, MIA was found to have the closest relationship with IAC (correlation: 0.96, distance: 89.88), highlighting their ability to breach the basement membrane and acquire metastatic potential.

In addition, analysis of sample‐to‐sample Euclidean distances between LUAD subtypes and healthy controls revealed a progressive increase in epigenetic divergence from AIS to IAC (Figure 6E). This trend may quantitatively demonstrate the cumulative effect of epigenetic alterations during tumour progression. Remarkably, the median Euclidean distance for healthy‐AAH comparisons exceeded that of AIS and approached MIA, potentially reflecting the heterogeneity of AAH samples. Examination of H3K4me3 enrichment patterns for five selected MERGEs (KDM4C, OXSR1, RAD17, RUNX1 and NPR3) across pathological stages revealed distinct profiles between early (AAH and AIS) and later (MIA and IAC) stages (Figures 6F and S7). This suggests their potential involvement in tumour progression and invasion.

## 4 DISCUSSION

Our study primarily elucidated the regulatory relationships between epigenetic modifications and their impact on fragmentomic features in real‐world clinical cohorts. The identification of epigenetically regulated genes formed the critical foundation for the cfDNA fragmentomics‐based machine learning model. More importantly, the ensemble model achieved excellent sensitivity for patients with MIA and stage I disease when validated on an independent external cohort, underscoring its great potential for clinical translation in the early detection of lung cancer.

Lung cancer, particularly early‐stage non‐small cell lung cancer (NSCLC), often exhibits one of the lowest ratios of ctDNA in cfDNA compared with other cancer types.^47^ While previous studies have shown that ctDNA is enriched in shorter fragments within mono‐ and di‐nucleosomes,^48^, ^49^ it is important to note that significant enrichment of cfDNA fragments shorter than 150 bp was not observed in our lung cancer cohort. This underscores the necessity for even more detailed analyses to detect subtle differences.

Our finding shows that the subtle differences appear at near‐nucleosome scale, much finer than previous genome‐level, arm‐level or 5Mb bin analyses.^12^, ^50^ Recent papers have also explored gene‐level fragmentomics and their relationship to tissue gene expression.^12^, ^15^, ^16^, ^17^ However, knowledge derived from tissue samples does not always translate seamlessly to blood due to the complex processes of digestion and mixing.^51^ Therefore, integrating fragmentomic data with multi‐modal epigenomic information provides an innovative strategy for boosting the diagnostic capabilities of lung cancer. The combination of cfChIP‐seq, cfRRBS and lpWGS in this study was carefully chosen to accurately correspond to the three most extensively studied areas of epigenomics, including DNA methylation, histone modifications and chromatin accessibility.^52^ These three epigenomic layers target the same molecular entity, the cell‐free nucleosome complex, offering a unique opportunity to capture the complementary and interconnected aspects of gene regulation and chromatin dynamics in cfDNA biology. This integration enables the exploration of cancer‐specific signals with unprecedented depth and precision.

We found that cancer‐upregulated genes marked by cell‐free epigenomes contribute more significantly to ctDNA fragmentomic identity. These genes exhibited a substantially higher proportion of short fragments, DNase1 origin motifs and cancer‐specific motifs compared with downregulated genes, suggesting that they undergo stronger digestion and are closely associated with cancer‐related DNase activities. Building on previous research on the stepwise process of cfDNA fragmentation,^43^ our findings imply that the characteristic features of ctDNA are primarily shaped by extracellularly circulating DNASE1.

We exploited the opposing effects of H3K4me3 with DNA methylation^53^ and chromatin accessibility^15^ on gene expression in our method. Our results confirmed that cfDNA H3K4me3 modifications inversely correlate with DNA methylation and NDR. DNA methylation serves as a stable epigenetic marker, rich in lineage information but limited in capturing transient expression changes.^54^ In contrast, H3K4me3 reflects more dynamic regulatory patterns, not always aligning with methylation or NDR. Thus, our analysis revealed a limited number of genes consistently exhibiting the expected regulatory relationships across all three omics layers in the MERGEs.

The H3K4me3 cfChIP‐seq analysis revealed a progressive decline in genes activity across LUAD stages (from AAH to IAC), corresponding with reports of increasing promoter methylation abnormalities during precancerous progression.^55^ Early global hypomethylation may drive chromosomal activity and tumour immune microenvironment changes.^56^, ^57^ MERGE revealed candidate genes, including novel Nkx‐family activators Nkx6.2, is likely reinforcing NKX motif activity early in tumour progression.^58^ Additionally, RUNX1 alterations were observed significantly during AAH to IAC progression, suggesting RUNX1/2 activation initiates ECM protein expression, fostering an EMT niche in LUAD.^58^ This will help advance our understanding of lung cancer stratification and facilitate the discovery of systemic biomarkers for anti‐tumour drugs. Our study is the first to investigate the progression of LUAD through dynamic H3K4me3 changes in cfDNA, highlighting how these changes, in conjunction with genomic alterations, may drive cancer progression.^59^, ^60^ Therefore, H3K4me3 profiling holds promise for predicting disease progression, and our technique could complement existing cancer detection methods, particularly for early‐stage cancer with low tumour burden.

Despite progress in early detection of lung cancer through cfDNA mutational analysis, its clinical application remains challenging due to limited sensitivity.^61^ Several studies using cfDNA methylation or fragmentation‐based approaches have shown only mild improvements in sensitivity, which are still insufficient for clinical use, particularly for stage I cases that benefit most from early detection.^18^, ^19^, ^50^ Both our previous study and several others have suggested that sensitivity typically hovers around 60% when modelling with single‐omics data.^12^, ^62^ One of the key reasons for that is the inherent noise and fluctuations in single‐marker analyses, a challenge that becomes particularly pronounced due to the low cfDNA abundance in early‐stage cancer.^5^ The cleavage and fragmentation of cfDNA are influenced by nucleosome organisation, chromatin accessibility and disease status, rather than being random processes.^7^, ^10^, ^28^, ^50^ Our study leveraged their synergistic roles in gene regulation, with H3K4me3 and DNA methylation having opposing effects on gene expression^53^ and influencing nucleosome stability.^15^, ^46^ Therefore, the integration of epigenetic and fragmentomic characteristics can exclude possible confounding factors and identify cancer‐related features. Previous studies have confirmed that specific genomic features of cfDNA can provide more precise information about cancer compared with the whole genome.^7^ In this study, the superior discrimination in MERGE‐based models and the excellent sensitivity of the ensemble model in early‐stage lung cancer together illustrated the rationality and importance of our modelling strategy.

Considering the insidious onset and substantial disparities in prognosis between early‐stage and advanced lung cancer, the efficacy of detection model in early‐stage lung cancer should be particularly concerning. At a specificity of 83.1%, our model achieved a sensitivity of 95.1% for stage I lung cancer and 96.2% for MIA in the validation set, outperforming other reported early detection models.^63^, ^64^, ^65^ Notably, the validation set included a higher proportion of early‐stage disease, with 81.8% of patients at stages 0 and I compared with 61.9% in the training set, demonstrating the broad generalisability of the model. Additionally, AIS and AAH were excluded from almost all similar studies, likely due to a lack of detectable signals in the blood.^18^ It is noteworthy that 75% of AIS cases were correctly classified and 70% of AAH cases scored above the cutoff in the validation set. More intriguingly, neither AAH nor AIS were included in the training set, suggesting that the findings were objective and our model could offer critical insights into the very early events of tumourigenesis. The estimated 10‐year postoperative disease‐specific survival rates for both AIS and MIA cases were 100%.^8^ Furthermore, two recently published large randomised controlled studies have provided high‐level evidence for the non‐inferior outcomes of sublobar resection compared with lobectomy for small‐sized lung cancer.^66^, ^67^ Taken together, the combination of our model and advances in surgical techniques collectively provided an avenue to achieve a radical cure for early‐stage lung cancer without excessive loss of lung function.

Significant reductions in cost, along with increased accessibility and standardisation are prerequisites for the wide application of early detection model in clinical practice. Some groups have attempted to improve the model performance by incorporating features from different sequencing platforms or methods, resulting in a considerable increase in costs.^28^, ^65^ Similarly, integrating radiological features from CT images identified by radiologists in several studies was manpower‐consuming and experience dependent.^68^, ^69^ In marked contrast, epigenomic sequencing data in this study were employed to ensure the rationality of the proposed model, while the final fragmentomics‐based model used only the lpWGS data for cost effectiveness and convenience. Therefore, our model provided a feasible solution for implementing early detection of lung cancer in clinical practice.

Some limitations existed in the present study. Although we identified the regulatory relationships between epigenetic modifications and their impact on fragmentation features, the underlying mechanism remains undefined. Future in‐depth molecular biological studies are required to fully address this question. Since all participants in the current study were Asian, the generalisability of the model to non‐Asian populations remains uncertain due to the distinct mutational landscape observed in female non‐smoker Asian patients with LUAD. Further studies with more diverse tumour phenotypes and non‐Asian populations are needed. Given the profound differences between small cell lung cancer and NSCLC in terms of biological features and clinical management, the role of cfDNA fragmentomics‐based machine learning model in their differential diagnosis warrants further investigation to augment its clinical utility. Moreover, sample bias cannot be excluded owing to the relatively small sample size of AAH and AIS cases. Our findings of this study should be confirmed in larger studies in this patient population.

In conclusion, we demonstrated that lung cancer‐related changes in fragmentomic features were unevenly distributed across the genome, with significant enrichment in epigenetically regulated regions of specific genes. The cfDNA fragmentomics‐based model exhibited superior detection capacity, particularly for lung cancer at the very early stage. Our study provides an accurate and cost‐effective approach for the early detection of lung cancer and paves the way to improved patient outcomes.

## AUTHOR CONTRIBUTIONS

Hefei Li, Xueguang Sun and Naixin Liang designed and supervised the study. Yadong Wang, Qiang Guo, Zhicheng Huang, Haibo Wang, Bowen Li, Daoyun Wang, Bin Zhou, Chao Guo, Yuan Xu, Yang Song, Zhibo Zheng, Zhongxing Bing, Hefei Li, Xiaoqing Yu, Ka Luk Fung, Heqing Xu, Jianhong Shi, Meng Chen and Shanqing Li contributed to clinical information collection. Liyang Song and Chen Zhu performed the experiment. Yadong Wang, Liyang Song, Fei Zhao and Tiantian Gu performed technical development, data analysis and figure preparation. Haoxuan Jin, Shiyuan Tong, Sibo Zhu and Chen Zhu provided technical support. Liyang Song, Fei Zhao, Yadong Wang and Tiantian Gu drafted the manuscript. Yadong Wang, Liyang Song, Fei Zhao, Tiantian Gu, Haoxuan Jin, Shiyuan Tong, Sibo Zhu, Jinlei Song and Jing Liu contributed to the revision. Liyang Song arranged figures and drew illustrations. Tiantian Gu and Shuai Hong contributed to project administration. All authors had full access to all the data in the study, discussed the results and accepted the responsibility to submit the final manuscript for publication. All authors have read and approved the final version of the manuscript.

## CONFLICT OF INTEREST STATEMENT

L. S., F. Z., T. G., S. H., H. J., S. T., S. Z., C. Z., J. S., J. L. and X. S. are employees of Shanghai Weihe Medical Laboratory Co., Ltd, Shanghai, China. All other authors have declared no conflicts of interest.

## CONSENT FOR PUBLICATION

Not applicable.

### ETHICS STATEMENT AND CONSENT TO PARTICIPATE

The study was conducted in accordance with the Declaration of Helsinki and approved by the ethics committee of Affiliated Hospital of Hebei University (Approval No. HDFYLL‐IIT‐023‐005) and Peking Union Medical College Hospital (Approval No. I‐23PJ1205). Written informed consent was obtained from all enrolled participants prior participation.

## Supporting information



Supporting Information

Supporting Information

Supporting Information

Supporting Information

Supporting Information

Supporting Information

Supporting Information

Supporting Information

Supporting Information

Supporting Information

## Data Availability

The data supporting the findings of this study have been deposited in Genome Sequence Archive (Genome Sequence Archive for Human in BIG Data Center, Beijing Institute of Genomics, Chinese Academy of Sciences) under accession number PRJCA033843. This study does not employ any new algorithms, and the code used for statistical analysis is available on request from the corresponding author Naixin Liang.
