# Supplementary material for: Cell‐free epigenomes enhanced fragmentomics‐based model for early detection of lung cancer
Source: Clin Transl Med. 2025 Feb 5;15(2):e70225. doi: 10.1002/ctm2.70225 (PMC11798665; doi:10.1002/ctm2.70225)
Supplement: Supplementary file 1 — Supporting Information [file CTM2-15-e70225-s009.docx]

**Supplementary figures for**

**Cell-free Epigenomes Enhanced Fragmentomics-Based Model for Early Detection of Lung Cancer**

**Clinical and Translational Medicine (submitted in 2024)**

Yadong Wang^1#^, Qiang Guo^2#^, Zhicheng Huang^1#^, Liyang Song^3#^, Fei Zhao^3#^, Tiantian Gu^3^, Zhe Feng^4^, Haibo Wang^2^, Bowen Li^1^, Daoyun Wang^1^, Bin Zhou^2^, Chao Guo^1^, Yuan Xu^1^, Yang Song^1^, Zhibo Zheng^1^, Zhongxing Bing^1^, Haochen Li^1^, Xiaoqing Yu^1^, Ka Luk Fung^1^, Heqing Xu^1^, Jianhong Shi^5^, Meng Chen^5^, Shuai Hong^3^, Haoxuan Jin^3^, Shiyuan Tong^3^, Sibo Zhu^3^, Chen Zhu^3^, Jinlei Song^3^, Jing Liu^3^, Shanqing Li^1^, Hefei Li^2*,^ Xueguang Sun^3*^, Naixin Liang^1*^

^1^Department of Thoracic Surgery, Peking Union Medical College Hospital, Chinese Academy of Medical Sciences and Peking Union Medical College, Beijing, China.

^2^Department of Thoracic Surgery, Affiliated Hospital of Hebei University, Baoding, China.

^3^Shanghai Weihe Medical Laboratory Co., Ltd, Shanghai, China.

^4^Department of Cardiothoracic Surgery, the Sixth Hospital of Beijing, Beijing, China.

^5^Department of Scientific Research, Affiliated Hospital of Hebei University, Baoding, China.

**Supplementary Information include:**

7 supplementary figures with legends

**
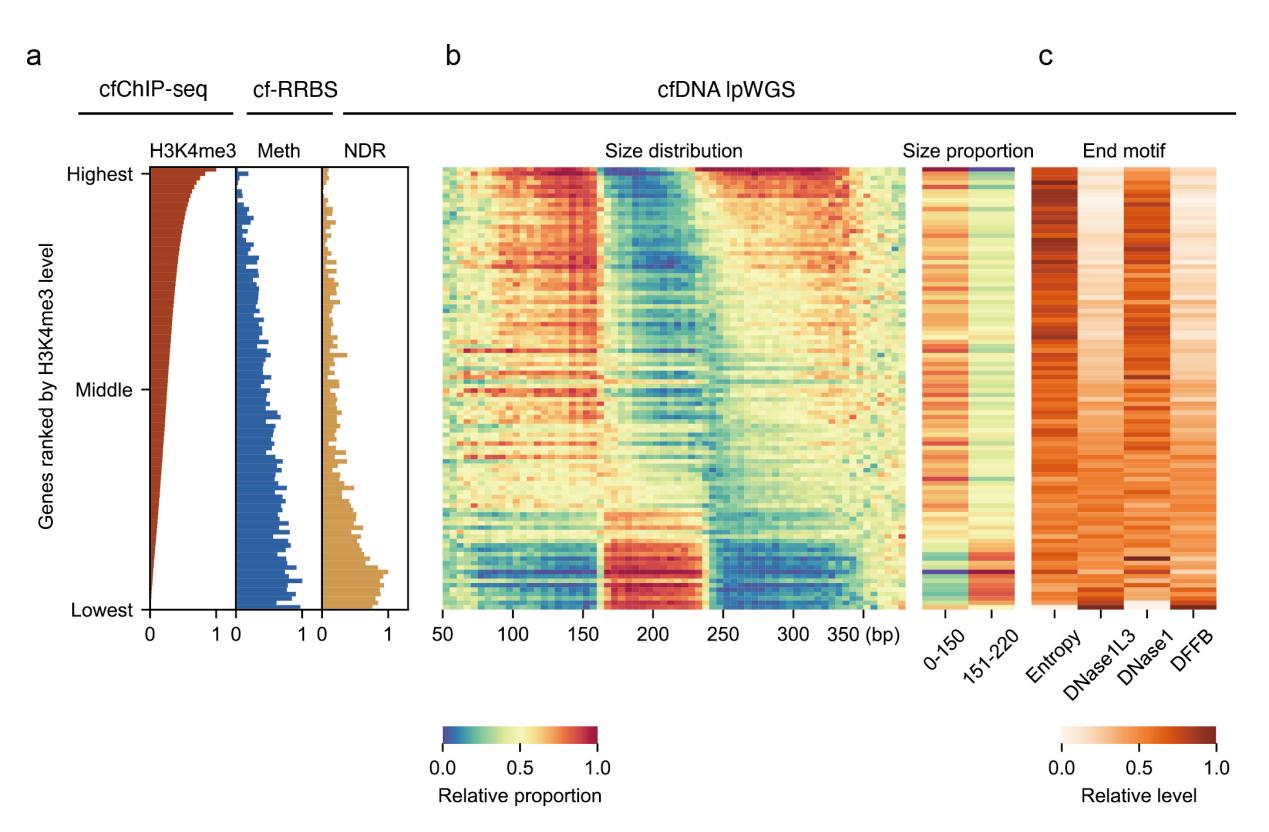
**

**Supplementary Fig. 1 Correlations between multiple cell-free epigenomes and fragmentomic characteristics in patients with lung cancer.** a-c The analysis of cell-free epigenomes and fragmentomic features, similar to Fig. 2, was performed on cancerous samples in the training set instead of healthy samples. Each row represents genes ranked by H3K4me3 levels, from highest to lowest, based on cfChIP-seq data and grouped into 100 percentiles. cfChIP-seq, cell-free chromatin immunoprecipitation sequencing; cfRRBS, cell-free reduced representation bisulfite sequencing; lpWGS, low-pass whole-genome sequencing; NDR, nucleosome-depleted regions.


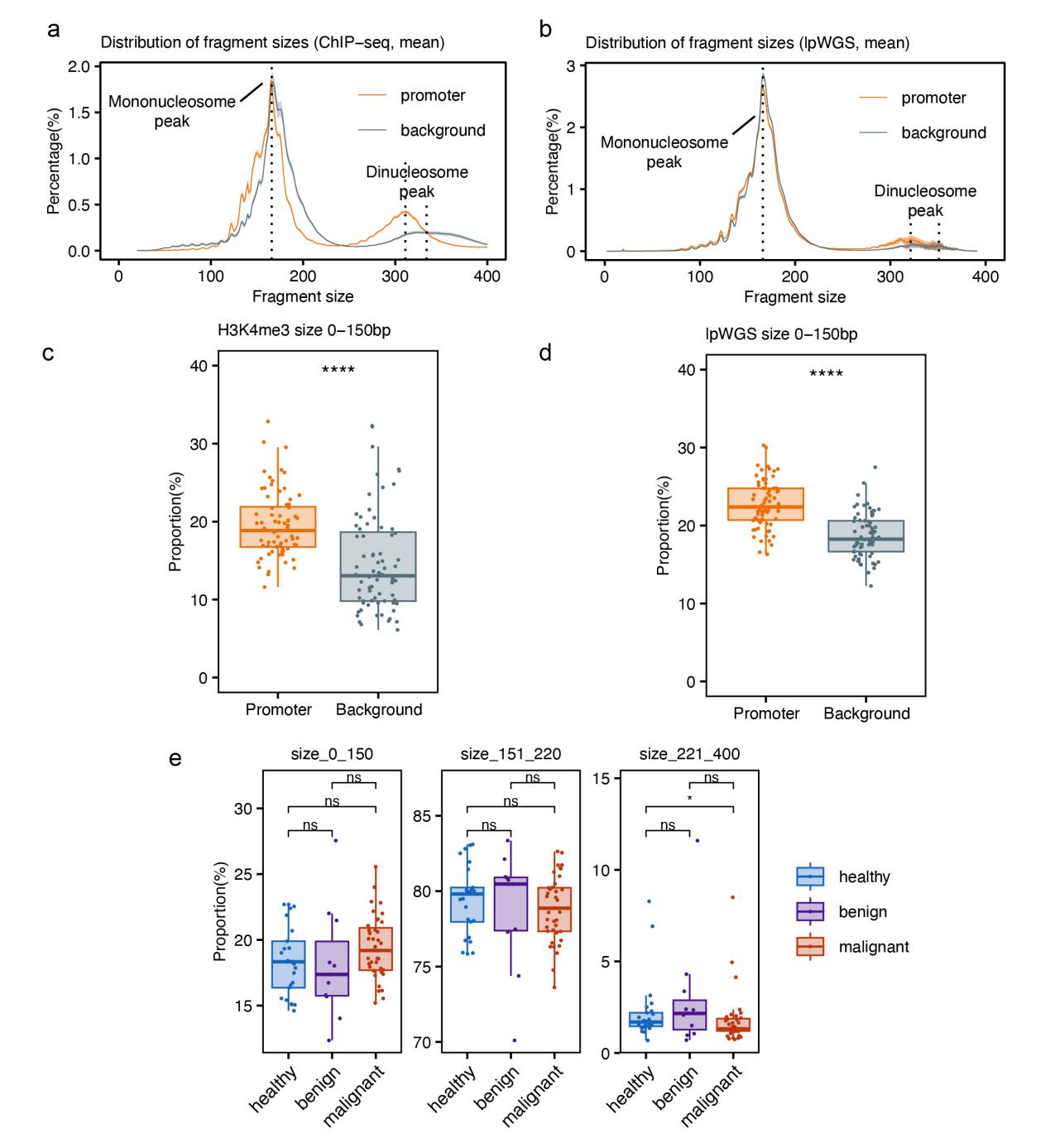


**Supplementary Fig. 2 Fragment size distributions of cfChIP-seq and lpWGS.** a and b Size distributions of fragments in promoter and promoter-depleted whole genome background of cfChIP-seq (a) and lpWGS (b), calculated in all training set samples. c and d Proportion of 0~150 bp fragment in promoter versus promoter-depleted whole genome background, calculated in ChIP-seq (c) and lpWGS data (d). e The relative proportions of fragment sizes across different groups, categorizing them into 0-150 bp, 151-220 bp, and 221-400 bp, based on lpWGS data. cfChIP-seq, cell-free chromatin immunoprecipitation sequencing; lpWGS, low-pass whole-genome sequencing. Asterisks indicate statistical significance (p-value were computed with Mann-Whitney U rank sum test, ns: p > 0.05, *: p < 0.05, **: p < 0.01, ***: p < 0.001, ****: p < 0.0001).


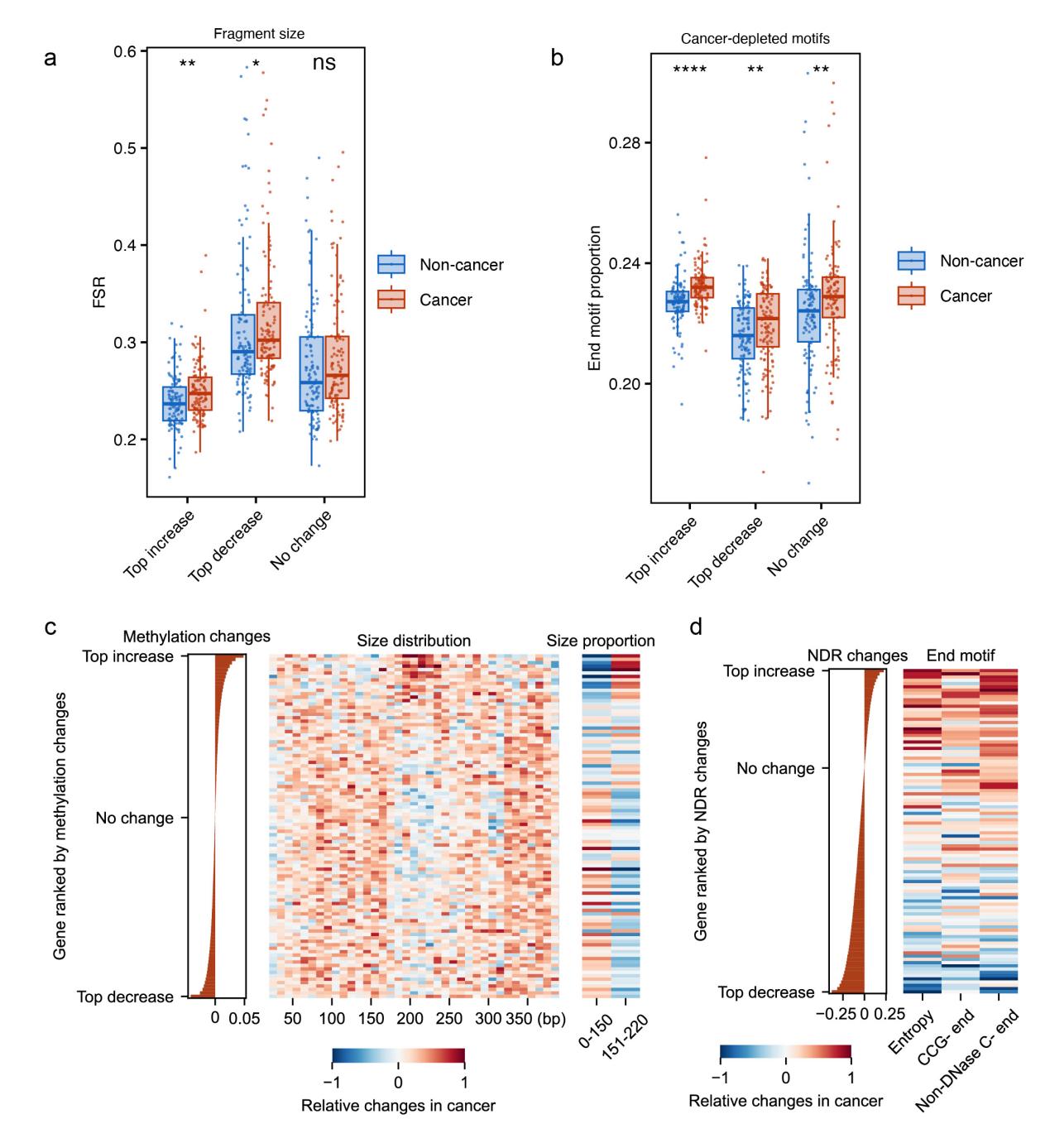


**Supplementary Fig. 3 Cancer-derived fragment characteristics correlated with multiple epigenetic regulation.** a FSR levels were calculated for the top 1% of genes with increased, decreased, and minimal changes in H3K4me3 levels between cancer and non-cancer samples. b End motif proportions of cancer-enriched motifs were calculated for the top 1% of genes with increased, decreased, and minimal changes in H3K4me3 levels between cancer and non-cancer samples. Cancer-enriched or depleted 4-mer motifs were identified with an adjusted p-value of < 0.05 between cancer and non-cancer samples. c and d Each row represent genes ranked by changes in DNA methylation or NDR levels, from increase to decrease, grouped into 100 percentiles. The heatmap in the middle shows fragment size distribution (10 bp windows). Colors and bar heights were Z-score scaled column-wise to a range of -1 to 1 to show relative changes. FSR, fragmentation size ratio; NDR, nucleosome-depleted regions. Asterisks indicate statistical significance (p-value were computed with Mann-Whitney U rank sum test, ns: p > 0.05, *: p < 0.05, **: p < 0.01, ***: p < 0.001, ****: p < 0.0001).

**
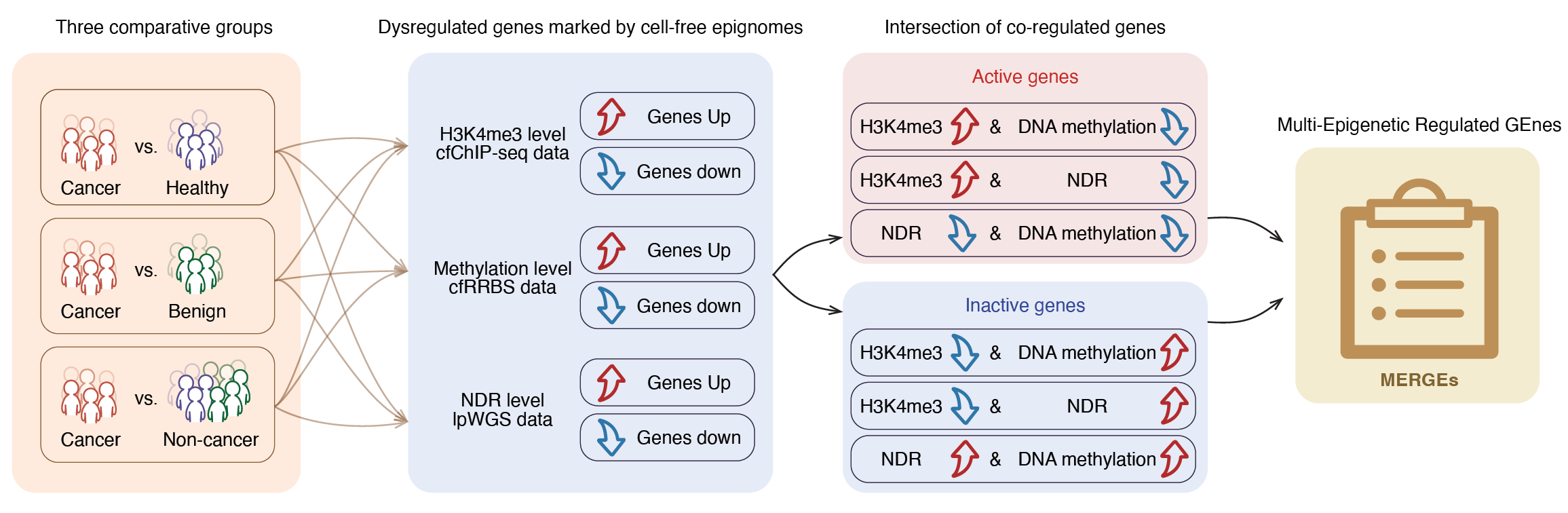
**

**Supplementary Fig. 4 The scheme for screening active and deactive multi-epigenetically regulated genes (MERGEs) against three different control groups.**


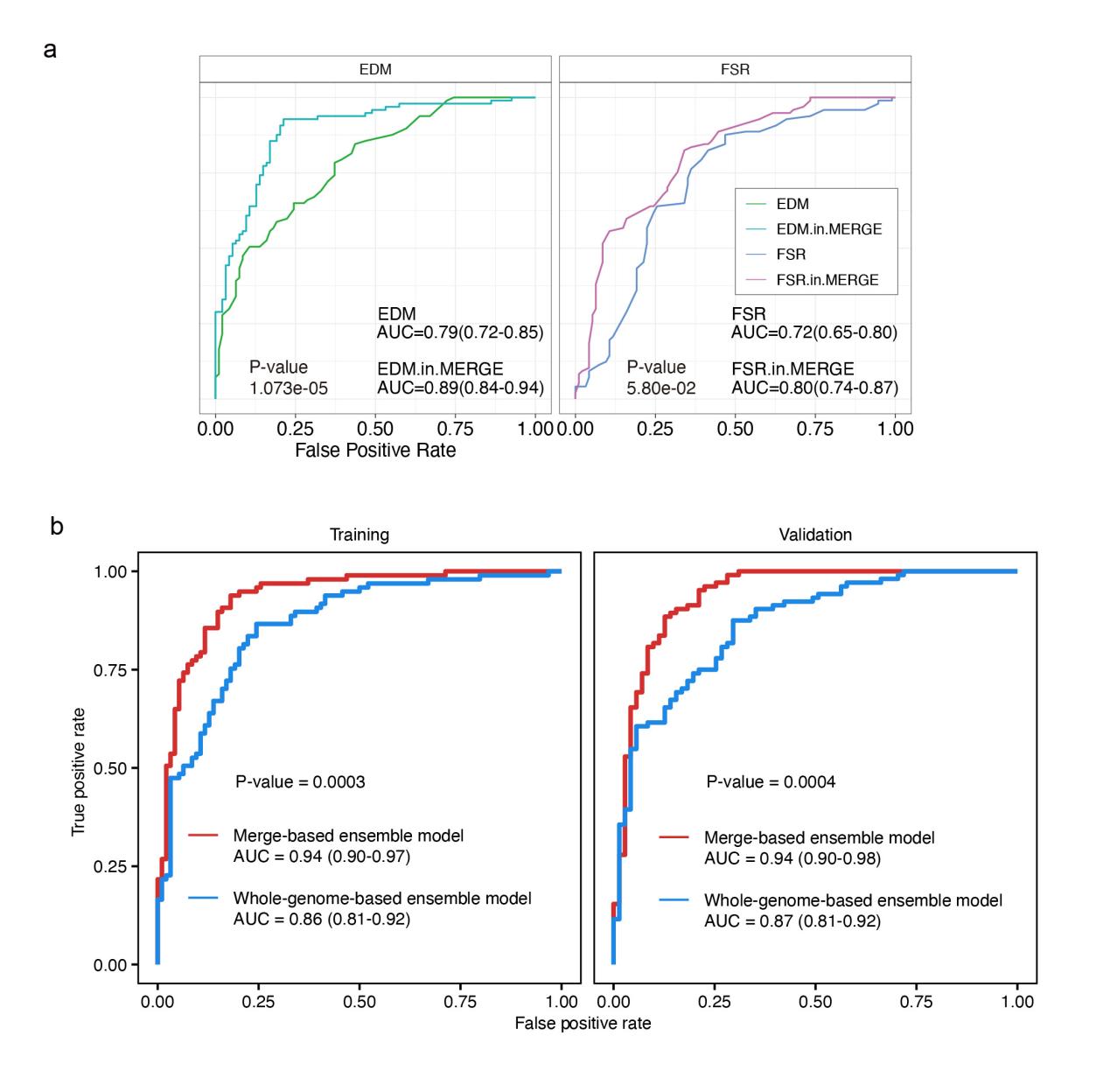


**Supplementary Fig. 5 Comparisons between the MERGE-based model and the whole genome-based model for lung cancer detection.** a Receiver Operating Characteristic (ROC) curve evaluating the performance of EDM and FSR in distinguishing cancer from non-cancer subjects in the training set. b ROC curve evaluating the performance of the MERGE-based ensemble model and the whole genome-based ensemble model in the training and validation set. EDM, end motifs; FSR, fragmentation size ratio; MERGE, multi-epigenetically regulated genes.


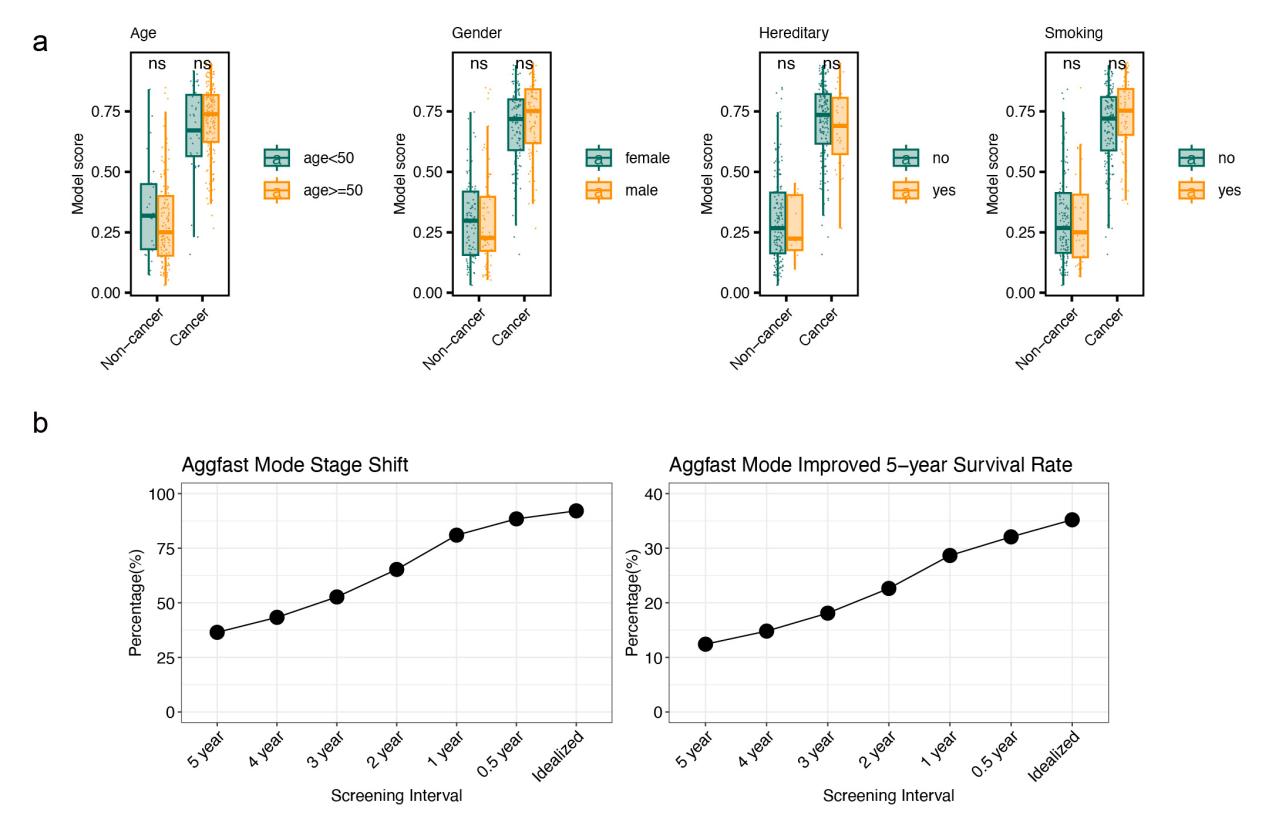


**Supplementary Fig. 6 The robustness of predictive model and estimation of stage shift benefit.** a The distribution of model scores across age, gender, hereditary status, and smoking status subgroups. b Estimation of the potential benefit of the MERGE-based ensemble model across different testing intervals, ranging from 5 years to continuous (idealized) testing. MERGE, multi-epigenetically regulated genes. Asterisks indicate statistical significance (p-value were computed with Mann-Whitney U rank sum test, ns: p > 0.05, *: p < 0.05, **: p < 0.01, ***: p < 0.001, ****: p < 0.0001).

**
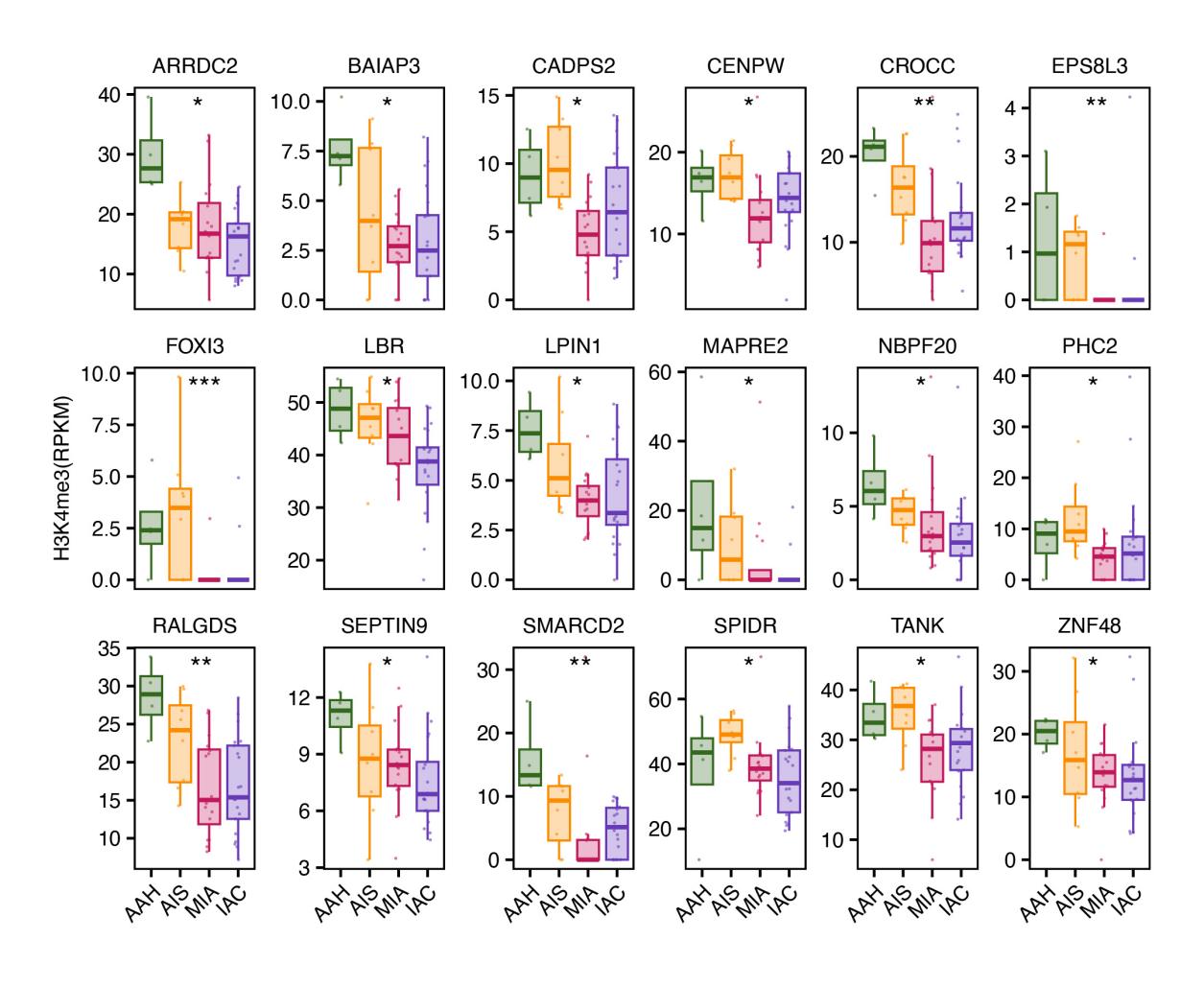
**

**Supplementary Fig. 7 Box plots show H3K4me3 enrichment levels of genes at different stages of lung adenocarcinoma progression.** Asterisks indicate statistical significance (p-value were computed with Mann-Whitney U rank sum test, ns: p > 0.05, *: p < 0.05, **: p < 0.01, ***: p < 0.001, ****: p < 0.0001).).
